# The Influence of Circadian Rhythms on Transcranial Direct Current Stimulation (tDCS) Effects: Theoretical and Practical Considerations

**DOI:** 10.3390/cells14151152

**Published:** 2025-07-25

**Authors:** James Chmiel, Agnieszka Malinowska

**Affiliations:** 1 Faculty of Physical Culture and Health, Institute of Physical Culture Sciences, University of Szczecin, Al. Piastów 40B Block 6, 71-065 Szczecin, Poland; 2 Institute of Psychology, University of Szczecin, 71-017 Szczecin, Poland

**Keywords:** tDCS, transcranial direct current stimulation, circadian rhythm, non-invasive brain stimulation, neurostimulation, neuromodulation

## Abstract

Transcranial direct current stimulation (tDCS) can modulate cortical excitability in a polarity-specific manner, yet identical protocols often produce inconsistent outcomes across sessions or individuals. This narrative review proposes that much of this variability arises from the brain’s intrinsic temporal landscape. Integrating evidence from chronobiology, sleep research, and non-invasive brain stimulation, we argue that tDCS produces reliable, polarity-specific after-effects only within a circadian–homeostatic “window of efficacy”. On the circadian (Process C) axis, intrinsic alertness, membrane depolarisation, and glutamatergic gain rise in the late biological morning and early evening, whereas pre-dawn phases are marked by reduced excitability and heightened inhibition. On the homeostatic (Process S) axis, consolidated sleep renormalises synaptic weights, widening the capacity for further potentiation, whereas prolonged wakefulness saturates plasticity and can even reverse the usual anodal/cathodal polarity rules. Human stimulation studies mirror this two-process fingerprint: sleep deprivation abolishes anodal long-term-potentiation-like effects and converts cathodal inhibition into facilitation, while stimulating at each participant’s chronotype-aligned (phase-aligned) peak time amplifies and prolongs after-effects even under equal sleep pressure. From these observations we derive practical recommendations: (i) schedule excitatory tDCS after restorative sleep and near the individual wake-maintenance zone; (ii) avoid sessions at high sleep pressure or circadian troughs; (iii) log melatonin phase, chronotype, recent sleep and, where feasible, core temperature; and (iv) consider mild pre-heating or time-restricted feeding as physiological primers. By viewing Borbély’s two-process model and allied metabolic clocks as adjustable knobs for plasticity engineering, this review provides a conceptual scaffold for personalised, time-sensitive tDCS protocols that could improve reproducibility in research and therapeutic gain in the clinic.

## 1. Introduction

Circadian rhythms are intrinsic biological processes that regulate a number of physiological and behavioural functions in living organisms [1,2], including humans [3]. These rhythms roughly follow a 24 h cycle and play a fundamental role in coordinating processes such as sleep–wake patterns, hormone secretion [4], body temperature [5], and metabolism [6]. Furthermore, emerging evidence suggests that circadian rhythms also influence brain function and cognitive processes [7]. These roles also include an impact on human health, and there is a two-way relationship at play. Disruptions to circadian rhythms can worsen the severity of diseases [8], and, at the same time, illnesses have the potential to disturb normal circadian rhythms [9,10,11]. Emerging epidemiological data show that, when a person’s sleep–wake pattern is persistently misaligned with their internal circadian phase—whether through extreme evening chronotype, delayed sleep-phase disorder, or rotating shift work—global health risks rise substantially. Late chronotype has been linked to higher odds of major depressive disorder and anxiety [12,13], an elevated incidence of hormone-dependent and colorectal cancers [14], and greater prevalence or intensity of chronic pain conditions such as musculoskeletal pain [15]. Mechanistically, misalignment dampens melatonin signalling, perturbs circadian control of DNA-repair genes, increases systemic inflammation, and disrupts endogenous opioid and mono-aminergic tone—pathways implicated in tumorigenesis, mood regulation, and nociception [16,17,18].

### 1.1. Borbély’s Two-Process Model of Sleep Regulation

The contemporary two-process model of sleep regulation is based on animal and human studies. These studies show that sleep probability and intensity depend on two factors: the superposition of a homeostatic drive, Process S, whose magnitude is proportional to prior wakefulness, and a circadian drive, Process C, generated by the suprachiasmatic pacemaker [19,20]. The first evidence originated from long-term electroencephalographic telemetry in rats. Borbély and Neuhaus showed that low-frequency non-REM (NREM) activity (“slow waves”) declined progressively during the light phase but rebounded after 12 h or 24 h of enforced wakefulness [21]; when the 24 h deprivation concluded at dark onset—the rodent’s behavioural activity phase—the rebound was split into two distinct waves, revealing that the rising sleep pressure was gated by the circadian rest–activity rhythm [21]. Subsequent lesion experiments established that this rebound persisted in animals lacking an intact suprachiasmatic nucleus, proving that the recovery mechanism—later formalised as Process S—does not require the circadian oscillator [22].

Translation to humans followed rapidly. A sleep-deprivation protocol combined with all-night spectral analysis demonstrated that EEG slow-wave activity (SWA) within successive NREM-REM cycles decays according to a single exponential function whose initial value is dictated by the duration of prior wake, thereby operationalising Process S as an exponential saturating/decaying variable [23]. In parallel, vigilance rhythms recorded during prolonged wake furnished a sinusoidal template for Process C [24,25]. The interaction of the two processes was codified in Borbély’s 1982 [19] synthesis, in which total sleep propensity equals S + C, and awakenings arise at the intersection of a declining S with a circadian-modulated arousal threshold. Daan, Beersma, and colleagues added an upper “sleep-onset” threshold, so that Process S oscillates between two circadian rails like a somnostat, enabling quantitative simulations of nap protocols, bed-rest studies, and internal desynchrony without invoking multiple oscillators [20]. The unambiguous separation of the two drives was confirmed a decade later: under a forced-desynchrony schedule that dissociates the sleep–wake cycle from the pacemaker, both sleep architecture and waking performance exhibited quasi-equal contributions of S and C, whereas SWA remained predominantly homeostatic; moreover, when homeostatic pressure was high, the circadian amplitude of waking virtually disappeared, illustrating a non-linear interaction [26].

Markers for the two processes are now firmly established. SWA in NREM sleep and theta power during quiet waking tracks the rise and decay of Process S [27], whereas melatonin secretion, core temperature, and locomotor activity report Process C [28,29]. Crucially, sleep homeostasis survives SCN ablation, underscoring the anatomical independence of the two controllers [30].

### 1.2. Synaptic Homeostasis Hypothesis

Another prominent theory, the Synaptic Homeostasis Hypothesis (SHY), posits that wakefulness broadly potentiates synapses, while sleep serves to down-scale synaptic strength and restore neural plasticity capacity [31]. In other words, prolonged wakefulness (high Process S) leads to net synaptic potentiation, and sleep reverses this by globally depressing synaptic connections to prevent saturation [32]. Animal studies have demonstrated molecular and electrophysiological signs of synaptic strengthening after extended wakefulness and synaptic renormalisation after sleep [33,34,35,36,37], consistent with SHY. This sets up the rationale that both circadian timing and sleep history can influence tDCS outcomes. Direct, cellular-level support for SHY has just been provided by Suppermpool et al., who longitudinally imaged every excitatory synapse on single zebrafish tectal neurons across complete sleep–wake cycles [38]. Synapse numbers rose during spontaneous or enforced wake and fell during ensuing sleep but only when homeostatic sleep pressure was high; low-pressure daytime sleep failed to drive down-scaling unless adenosine was pharmacologically boosted and noradrenergic tone suppressed. Thus, not all sleep epochs are equally ‘restorative’, and Process S—rather than sleep per se—gates synaptic renormalisation.

Notably, circadian influences are tightly intertwined with the sleep–wake cycle. An individual tested in the evening not only has a different circadian phase than in the morning but also a higher accumulated sleep drive (Process S) after a day of wakefulness. Both factors can elevate or dampen cortical excitability [39]. This interplay must be considered when optimising tDCS timing.

Transcranial direct current stimulation (tDCS) is a non-invasive brain stimulation technique that has garnered significant attention in recent years for its potential to modulate neural excitability [40], enhance cognitive functions [41], and promote brain plasticity [42] in a safe manner [43]. tDCS alters the excitability of the brain via a weak direct current (usually 0.5–2 mA) applied to the scalp [44]. Changes in excitability (facilitation vs. inhibition) depend on the polarity of the electrical current, allowing for anodal and cathodal stimulation [45,46]. Anodal tDCS increases cortical excitability, while cathodal tDCS typically inhibits it [46,47]. The neurobiological impacts of tDCS may extend beyond the stimulation duration, persisting for up to 90 min following a 15 min session [48], attributed to a modulating effect on N-methyl-D-aspartate (NMDA) receptors and GABAergic activity [49,50]. This method triggers calcium-dependent glutamatergic synaptic plasticity, inducing long-term depression (LTD) or long-term potentiation (LTP) [51].

While the efficacy of tDCS on various cognitive, behavioural, and emotional functions in both healthy and clinical populations has been extensively studied [52,53,54,55,56], less attention has been paid to the possible impact of circadian rhythms on the effects of tDCS and their magnitude. The timing of tDCS stimulation could interact with the natural fluctuations in circadian patterns in brain function, potentially affecting the outcome of the stimulation [57]. Therefore, understanding the relationship between tDCS and circadian rhythms is essential for optimising stimulation protocols and maximising overall effectiveness. It is worth noting that, while tDCS can be utilised to improve and enhance circadian rhythm factors (e.g., sleep quality, sleep disorders, circadian misalignment) [58,59,60], that aspect is beyond the scope of this review and will not be discussed.

This review proposes that tDCS produces robust and polarity-specific after-effects only when it is delivered inside a circadian–homeostatic “window of efficacy”.

1. On the circadian axis (Process C), the window opens during the late biological morning and early evening, when intrinsic alertness and membrane depolarisation peak.
2. On the homeostatic axis (Process S), the window is widest after consolidated sleep, when synaptic weights have been renormalised.
3. Outside this overlap—i.e., under high sleep pressure or during the circadian trough—baseline excitability is either saturated or too low, glucocorticoid tone is unfavourable, and tDCS effects are diminished or even reversed.

This framework integrates Borbély’s two-process model with synaptic-homeostasis data and correctly predicts the time-of-day reversals and sleep-loss failures repeatedly observed in human tDCS and TMS studies. The present article is a theoretical, narrative synthesis. This review does not aim to review all tDCS studies comprehensively but instead to synthesise key findings to demonstrate how circadian- and sleep-dependent states can modulate stimulation efficacy. This approach allows a mechanistic timing framework to be proposed without the rigid inclusion criteria of a systematic review.

### 1.3. Search Strategy, Inclusion Criteria, and Synthesis Approach

In this narrative review, we searched PubMed, Scopus, and Web of Science from database inception to 31 March 2025 using the combined terms (“tDCS” OR “transcranial direct current stimulation” OR “non-invasive brain stimulation”) AND (“circadian” OR “time of day” OR “chronotype” OR “sleep deprivation”). After removal of duplicates (n = 20), two authors independently screened titles and abstracts and examined 112 full-text articles. Inclusion criteria were (a) peer-reviewed original research; (b) human or mammalian animal models; (c) tDCS—or transcranial magnetic stimulation when excitability data could inform tDCS mechanisms—applied in relation to circadian phase, chronotype, or experimentally manipulated sleep; and (d) outcomes on cortical excitability, neuroplasticity, cognition, sleep, or health markers. Exclusion criteria were case reports (<5 participants), non-English papers, or studies lacking circadian-related variables. Eight studies fulfilled all criteria.

## 2. Circadian Rhythms and Brain Function, Metabolism, Body Temperature, Cortisol, and Neurotransmitter Systems

Circadian rhythms follow an approximate 24 h period synchronised with environmental cues of light and darkness, allowing organisms to adapt their internal physiology to the external day–night cycle [61]. The primary pacemaker responsible for orchestrating circadian rhythms in mammals is the suprachiasmatic nucleus (SCN) located in the hypothalamus [62]. The SCN receives direct input from specialised slow light change-sensitive retinal ganglion cells, which then relay information about light exposure to the brain [63]. This input allows the SCN to align the body’s internal clock with the external light–dark cycle, thus entraining circadian rhythms to daylight (natural or artificial). In the absence of external time cues, the SCN can closely maintain the rhythm of 24 h through its intrinsic oscillatory properties [64].

The thalamic intergeniculate leaflet (IGL) also plays a crucial role in the regulation of circadian rhythms, acting as an integral component of the circadian system alongside the SCN [65]. The IGL, identified in 1974 as a region projecting to the SCN [66], became the first site distal to the SCN acknowledged for its contribution to circadian system regulation [67]. It contains neuropeptide Y-IR cells and provides NPY terminals to the SCN [67], with studies demonstrating that NPY infused into the SCN can elicit circadian rhythm phase shifts [68]. The IGL’s connectivity to brain nuclei involved in visuomotor functions, sleep, and the orexin system suggests that it may act as a central hub for distributing rhythmically governed sleep regulatory information [67]. In addition to its role in circadian rhythm regulation, the IGL appears to contribute to the control of eye movements during sleep [67].

Circadian rhythms influence a wide array of physiological and behavioural processes, with the sleep–wake cycle one of the most notable examples. The SCN regulates the timing of sleep and wakefulness through its connections with the brain regions involved in sleep regulation [25]. One key region is the pineal gland, which secretes the hormone melatonin to promote sleepiness [69]. The superior cervical ganglion, whose postganglionic axons innervate the pineal gland, contributes to circadian rhythm through a connection that regulates melatonin production [70]. Additionally, neurons in the intermediolateral column of the spinal cord project to noradrenergic neurons in the superior cervical ganglion, stimulating the pineal gland to produce melatonin [71]. Moreover, it is also necessary to mention the role of the paraventricular nucleus, which is a critical regulator of rhythmic energy intake and metabolism [72].

Humans exhibit a pronounced circadian rhythm in cortisol secretion, peaking shortly after awakening in the early morning (the cortisol awakening response) and then declining throughout the day to reach a low point in the evening [73,74,75]. Cortisol is a potent neuromodulator that can rapidly affect synaptic function and neuronal excitability [76,77]. Fluctuations in cortisol across the day thus have significant impacts on brain function. Early-day high cortisol generally promotes alertness and baseline cortical excitability, helping to counteract sleep pressure during the “wake-maintenance zone” of the day [78]. For instance, one study using transcranial magnetic stimulation (TMS) and EEG found that intrinsic cortical excitability varies circadianly and is highest in individuals with the largest amplitude of circadian endocrine markers (like cortisol). In that study, cortical reactivity to TMS correlatively tracked the circadian phase: after an initial afternoon dip, cortical excitability rose overnight in parallel with the surge of cortisol toward its early-morning peak [78]. This suggests that cortisol’s daily rise is associated with increased neuronal responsiveness, potentially as part of the brain’s preparation for daytime cognitive demands.

However, chronically elevated or dysregulated cortisol is detrimental to neuroplasticity and cognition. Both unusually high and abnormally low cortisol levels (e.g., in Cushing’s or Addison’s disease, or due to chronic stress) can impair synaptogenesis and memory [79]. In older adults, a flatter diurnal cortisol profile or higher daily cortisol output correlates with worse performance in memory, processing speed, and other cognitive domains [80]. By contrast, a more dynamic, robust cortisol rhythm has been associated with better cognitive function [81]. Notably, morning cortisol should not be “too high”—excessive glucocorticoid activity can suppress long-term potentiation (LTP) and memory retrieval, whereas moderate physiological levels optimise them (an inverted-U effect) [76,82,83]. Thus, the normal circadian cortisol rhythm may enhance cognition by providing a surge of cortisol to energise the brain in the morning, followed by a gradual decline that permits optimal synaptic plasticity later in the day. Indeed, studies report that many cognitive functions (attention, working memory, recall) follow a circadian pattern, often peaking when cortisol is moderate or declining rather than at its zenith [84]. For example, Johnson et al. found that short-term memory and alertness both show circadian peaks in the midday and afternoon, paralleling the post-morning decline in cortisol and the rise in body temperature [85].

On a cellular level, cortisol’s effects on neurons are mediated by high-affinity mineralocorticoid and glucocorticoid receptors in the hippocampus and prefrontal cortex [86]. These receptors modulate gene expression and synaptic transmission. A robust morning cortisol pulse may prime neural circuits for the day’s activity (as some have theorised, the cortisol awakening response “prepares” the brain for anticipated demands) [87], but sustained high cortisol can trigger synaptic atrophy or inhibited plasticity [88,89]. In sum, circadian cortisol oscillations contribute to a daily ebb-and-flow in brain excitability and cognitive performance. High morning cortisol provides arousal and metabolic resources for brain function, while the afternoon/evening low cortisol state appears more favourable for certain forms of synaptic potentiation and memory encoding. This is supported by direct experimental evidence: when exogenous cortisol is administered to mimic a high-cortisol state at times when levels are normally low, it can blunt neuroplasticity. For instance, Sale et al. found that a form of associative plasticity (paired associative stimulation, PAS) was more effective in the evening (when endogenous cortisol is low) and that giving a dose of hydrocortisone at that time abolished the enhancement, implying cortisol elevation can suppress induced plasticity [90]. A complementary study showed that individuals have better motor cortex plastic responses on days when their morning cortisol surge is larger than usual—suggesting that an appropriately robust (but not excessive) cortisol rhythm facilitates neuroplastic readiness [91]. Together, these findings indicate that cortisol’s circadian rhythm modulates the brain’s excitability and capacity for plastic change across the day, likely through both direct receptor-mediated synaptic effects and indirect influences on metabolism, cerebral blood flow, and neurotrophin levels.

Core body temperature also follows a strong circadian pattern, reaching a nadir in the early morning (~2–4 AM, toward the end of the habitual sleep period) [92] and peaking in the late afternoon or early evening [93]. This ~1 °C fluctuation in brain and body temperature over 24 h has pronounced effects on neural function. Temperature profoundly influences neural activity and synaptic efficiency—warmer temperatures generally increase neuronal firing rates and conduction velocity, while cooler temperatures slow metabolic processes and can reduce excitability [94]. In fact, the brain’s master clock in the suprachiasmatic nucleus (SCN) drives daily rhythms in brain temperature, which serve as signals to modulate neural circuit behaviour and plasticity [94]. As a result, times of day with higher core temperature tend to correspond to higher arousal, faster cognitive processing, and possibly greater neuronal responsiveness, whereas the low-temperature phase is accompanied by sleepiness and reduced alertness.

Empirical studies in humans show that cognitive performance fluctuates in parallel with the body temperature rhythm. For example, short-term memory, attention, and reaction speed are worst in the early morning (when core temperature and vigilance are lowest) and improve significantly by late afternoon, around the temperature peak [85]. Subjective alertness follows a similar pattern, rising with increasing body temperature during the day. These effects are so robust that misalignment of the body temperature rhythm (e.g., in shift workers or jet-lag) reliably impairs cognitive functions like concentration and memory [95]. The physiological basis is that warmer body/brain temperature enhances enzymatic activity and neurotransmitter dynamics, effectively “warming up” neural circuits. Small changes (on the order of 1 °C) can alter the balance of ion channel kinetics and receptor function, thereby modulating excitability and synaptic efficacy. In one chronobiology analysis, constant ambient temperature manipulations demonstrated that maintaining a higher body temperature level can sustain better cognitive performance, reinforcing the link between the temperature rhythm and mental acuity [96].

Neuronal plasticity may also be subject to circadian temperature modulation. Experiments in animal models have found daily oscillations in the ease of inducing LTP or LTD in the brain, and it may be partly attributable to internal temperature and metabolism cycles [97]. Nocturnal increases in neuronal excitability (observed in some rodent and fly studies) have been linked to circadian changes in intracellular ion concentrations and temperature-dependent enzyme activity in synapses [97]. In the SCN itself, a higher brain temperature during the day helps sustain firing, while cooling at night reduces SCN neuron excitability—demonstrating how temperature rhythms drive neural activity rhythms [94]. Translating to humans, while direct evidence is limited, it is plausible that the late-day rise in core temperature creates a neurophysiological milieu more permissive to plastic changes. The evening peak in temperature coincides with peak alertness and has been associated with improved motor learning and cognitive throughput in some studies. By contrast, early morning (when temperature is lowest) is associated with grogginess and diminished cognitive flexibility. Indeed, the circadian trough of body temperature (pre-dawn) is a “vulnerable” period characterised by minimal cortical responsiveness and heightened sleep propensity, which is the opposite of an optimal state for learning or external stimulation.

Contrary to early “moving set-point” hypotheses, the circadian rise and fall are not merely passive reflections of behavioural activity or changes in a thermoregulatory set-point. Telemetry and gradient-chamber experiments show that mammals actively choose cooler ambient zones when their core temperature is high and warmer zones when it is low, indicating that the set-point remains constant and that the SCN-driven rhythm is imposed on, and partially opposed by, homeostatic thermoregulation [98]. The resulting waveform represents a subtle, orchestrated mismatch between metabolic heat production and heat loss.

It is important to note that core body temperature and cortisol rhythms are intertwined but distinct modulators. Under normal conditions, body temperature starts rising later in the day than cortisol and remains elevated into the evening when cortisol is low. Each rhythm influences the brain state via different mechanisms—cortisol via hormone receptors and genomic effects, and temperature via biophysical and metabolic effects. Both are crucial for maintaining the daily oscillation in brain excitability. For instance, the pronounced evening alertness (sometimes called the “evening peak” in cognitive performance) correlates with high body temperature and low cortisol—a combination that seems to favour neural network flexibility and efficient information processing. The morning, conversely, combines high cortisol (high arousal but potentially inhibiting plasticity) with low temperature (reduced neural throughput), which may explain why complex cognitive tasks or induction of neuroplastic changes can be less effective at dawn. Overall, circadian variations in core temperature modulate neuronal responsiveness and plasticity largely by aligning the organism’s peak performance period with the optimal internal thermal state.

Circadian timing shapes metabolism from gene transcription to whole-organ energy balance. In mouse liver—the best-charted tissue—≈20% of all expressed genes oscillate with 24 h periodicity, including rate-limiting enzymes for glycolysis, gluconeogenesis, lipid and cholesterol synthesis, and xenobiotic clearance. Rhythmic transcription is bio-energetically economical and temporally separates incompatible pathways, a control that collapses when animals are fasting continuously or carry circadian mutations but is amplified when the same calories are confined to an 8 to 12 h feeding window. Comparable-omics surveys reveal that 10% of genes oscillate in most peripheral organs, underscoring the ubiquity of metabolic clocks beyond the suprachiasmatic nucleus (SCN) [6].

Glucose tolerance and insulin action are distinctly diurnal: oral glucose is cleared best in the morning and worst in the evening in humans, a pattern mirrored by rodents whose active phase occurs at night. SCN ablation or autonomic denervation abolishes these rhythms, demonstrating central-clock control [6]. At the organ level, a “six-clock” network coordinates carbohydrate flux: the SCN sets systemic insulin sensitivity; gut and muscle clocks gate nutrient uptake; liver, adipose, and pancreatic clocks schedule gluconeogenesis, lipolysis, and insulin secretion, respectively.

Hepatic cholesterol conversion to bile acids peaks once per day through circadian expression of Cyp7a/b, while REV-ERB α rhythmically represses thermogenic UCP genes in brown adipose tissue; deleting Rev-erb α in BAT flattens both UCP expression and body-temperature rhythms [99]. Circulating non-esterified fatty acids also rise at night, reflecting clocked lipolysis in adipose depots [100].

Circadian clocks tune the brain’s chemical landscape on a 24 h schedule, regulating not only when neurotransmitters are released but also how they are synthesised, degraded, and perceived by their receptors.

Basal extracellular dopamine (DA) in dorsal and ventral striatum rises during the behavioural active phase of rodents and falls in the rest phase. This rhythm is generated locally: E-box elements in the promoters of the rate-limiting enzyme tyrosine hydroxylase, the dopamine transporter (DAT), and mono-amine oxidase-A are driven by clock-gene transcription factors, while the expression of Drd1-3 receptors is under the opposing control of ROR and REV-ERB nuclear receptors [101]. Conversely, DA feeds back on the clock—rhythmic D2-receptor activation gates daily peaks of the clock protein PER2 in dorsal striatum [101]—illustrating a bidirectional loop between catecholamine tone and molecular timekeeping.

Tryptophan hydroxylase, the rate-limiting enzyme in serotonin (5-HT) synthesis, oscillates in raphe neurons, yielding the highest 5-HT release into the SCN early in the night of nocturnal species, precisely when locomotor activity begins [102,103]. The SCN reciprocally projects to the raphe via the dorsomedial hypothalamus, creating a closed neural circuit. Rhythmic 5-HT in the SCN modulates light-induced phase shifts, and selective depletion or lesion of raphe pathways dampens behavioural rhythmicity, underscoring serotonergic entrainment of the master clock [102,103].

Clock-driven bursts of sympathetic noradrenaline from the superior cervical ganglion reach the pineal gland only at night, gating melatonin synthesis and reinforcing systemic time cues [104]. Wake-promoting histamine from tuberomammillary neurons peaks in the light (diurnal animals) or dark (nocturnal animals), mirroring the vigilance state, while histidine-decarboxylase mRNA shows the same phase-locked rhythm [104]. Orexin neurons follow a similar profile, with higher peptide content and cerebrospinal fluid levels in the active phase, linking arousal circuits to metabolic and motivational clocks [104].

Excitatory glutamate in the SCN rises in the late night, matching the timing of photic phase advances, whereas glutamate decarboxylase (GAD65) mRNA and extracellular GABA peak in antiphase during the day. Clock genes also sculpt receptor abundance: NR1/NR2B NMDA-receptor sub-units and AMPA-evoked currents display strong day–night variation within the SCN, contributing to time-of-day differences in light sensitivity and inter-SCN synchrony [104]. Outside the SCN, diurnal swings of dopamine, glutamate and GABA in striatum and nucleus accumbens coordinate reward processing with external time cues [104].

Cholinergic tone in cortex and hippocampus is higher during the active phase, supporting attention and learning, while diurnal histograms of acetylcholinesterase activity mirror this pattern [104,105]. Similar rhythmic signatures are documented for neuropeptides (e.g., VIP, AVP) that not only act as transmitters but also synchronise SCN cellular oscillators [105].

It should be noted that chronotype refers to an individual’s preferred timing of sleep–wake behaviours (i.e., “morning lark” vs. “night owl”), typically assessed via questionnaires (e.g., MEQ) or actigraphy. By contrast, circadian phase denotes the current position within a person’s ~24 h biological clock (e.g., melatonin onset, core body temperature nadir). While chronotype predicts habitual phase angle (whether melatonin onset falls earlier or later), circadian phase is the state at a given moment, which can be shifted by light exposure, sleep history, or travel.

Understanding the underlying mechanisms of circadian rhythms and their impact on cognitive functions allows us to develop time-sensitive interventions, such as tDCS. The following sections will explore aspects of brain and cognitive function under the influence of circadian rhythms that can affect tDCS behavioural and physiological results.

## 3. Circadian Modulation of Cognitive Functions

Circadian rhythms influence not only sleep–wake cycles but also play a crucial role in modulating various cognitive functions throughout the day. Several cognitive domains undergo circadian modulation, such as attention [106], memory [107], executive functions [108], and decision making [109]. Studies consistently demonstrate that cognitive performance is not uniform across the day and that certain cognitive processes peak at specific circadian phases. For example, attention, alertness, and faster reaction times tend to be highest during the morning hours, known as the “morning peak” [110], while memory consolidation and creative problem solving may peak in the afternoon or early evening [111]. It is also important to note that individual differences in circadian rhythms exist, leading to distinct chronotypes. Some individuals naturally exhibit a preference for morning activities and are labelled “morning types” or “larks,” while others prefer evening activities and are referred to as “evening types” or “owls.” These persons’ differences in chronotype are influenced by genetic factors and can impact an individual’s cognitive performance and alertness at different times of the day [112].

The intricate neurobiological mechanisms underpinning the circadian modulation of cognitive functions involve interactions between the SCN, brain regions responsible for cognitive processing, and the sleep–wake regulatory systems. The SCN’s influence on cognitive functions is mediated through its connections with other critical brain regions, such as the prefrontal cortex [113], hippocampus [114], and thalamus [115], which are essential for attention, memory, and executive functions [116].

The circadian system’s impact on cognitive performance is also closely related to the sleep–wake cycle. Adequate sleep is essential for optimal cognitive functioning, and the circadian timing of sleep plays a critical role in memory consolidation and cognitive restoration. Disruptions to the circadian rhythm, such as irregular sleep schedules or sleep deprivation, can result in impaired cognitive performance and reduced attentional resources [117]. In a study by Salehinejad et al. [118], the impact of sleep deprivation for one night versus a full night’s sleep on cognition and underlying brain physiology (e.g., cortical excitability and plasticity) was examined. They utilised transcranial magnetic stimulation (TMS) to stimulate the motor cortex and assess cortical excitability; applied tDCS to induce long-term potentiation (LTP)- and long-term depression (LTD)-like plasticity; and used behavioural tasks to measure motor learning, working memory, and attention. All participants engaged in two research sessions: one after a night of sufficient sleep (from 11:00 p.m. to 8:00 a.m.) and the other following sleep deprivation for a night during the same timeframe (11:00 p.m. to 8:00 a.m.). In summary, the study revealed that learning and memory formation, behavioural aspects of plasticity, and working memory and attention, which depend on cortical excitability, were compromised following one night of sleep deprivation.

In an earlier work, Salehinejad et al. [57] investigated how circadian preference (i.e., chronotype, a natural variation in circadian rhythms among humans) affects human cognition and underlying brain physiology. The research design involved recruiting participants with distinct morning and evening chronotypes, as determined by the Morningness–Eveningness Questionnaire (MEQ). The study also focused on balancing various confounding factors, such as gender and age, to ensure the reliability and validity of the findings. The researchers implemented multiple methodologies, including transcranial magnetic stimulation and tDCS, to monitor cortical excitability and induce neuroplasticity. They also employed several cognitive tasks to assess motor learning (SRTT), working memory (three-back letter task), and attentional functioning (Stroop task, AX-CPT). Additionally, the EEG recordings during the tasks provided insights into the neural correlates associated with behavioural performance. The experimental sessions were meticulously designed and standardised to take into account the participants’ sleep–wake cycles, light exposure, and other factors that could potentially influence cortical excitability. Moreover, the use of validated assessment tools and rigorous statistical analyses enhanced the scientific rigour of the study. They found that motor learning and cognitive performance (working memory and attention), along with their electrophysiological components, are significantly enhanced at the chronotype-aligned (phase-aligned) peak time, compared to the non-preferred time. This was associated with interesting changes in cortical excitability and tDCS-induced plasticity, which is discussed in the next section.

In another study by Wong et al. [119], the influence of time of day on tDCS-induced episodic memory retrieval was examined. In the fourth experiment relevant to this chapter, participants were randomly assigned to morning or afternoon sessions of tDCS. The results showed that tDCS over the dorsolateral prefrontal cortex (compared to a sham stimulation) improved recollection accuracy in the morning. This is consistent with research findings confirming that episodic memory performance is highest in the morning [120].

The results of the above four studies show that human cognition is hugely affected by circadian-related factors (e.g., sleep pressure, chronotype, time of the day, etc.). Accordingly, it is possible to pinpoint the most suitable stimulation times for specific cognitive domains. For instance, targeting attention and alertness with tDCS during the morning hours, when these functions are naturally enhanced, may yield more pronounced effects. Conversely, interventions aimed at memory consolidation or creative problem solving may be more effective during the afternoon or early evening [111]. Understanding the temporal dynamics of cognitive functions allows for the strategic application of tDCS. Morning and evening types may experience distinct cognitive advantages at different times of the day. Researchers can tailor stimulation protocols based on an individual’s chronotype, allowing for tDCS to be administered when cognitive functions are naturally at their peak for that person.

Furthermore, as the circadian system’s impact on cognitive performance is closely related to the sleep–wake cycle [117], optimising sleep patterns can further enhance the effects of tDCS. Adequate sleep and proper sleep timing are crucial for memory consolidation and cognitive restoration [121]. Therefore, integrating tDCS interventions with sleep optimisation strategies can provide synergistic benefits for cognitive enhancement. In any tDCS study targeting cognitive functions, it is crucial to assess the participants’ circadian rhythms and chronotypes. Tools such as questionnaires and actigraphy can help determine the timing of an individual’s peak performance to optimise intervention design.

**Figure 1:**
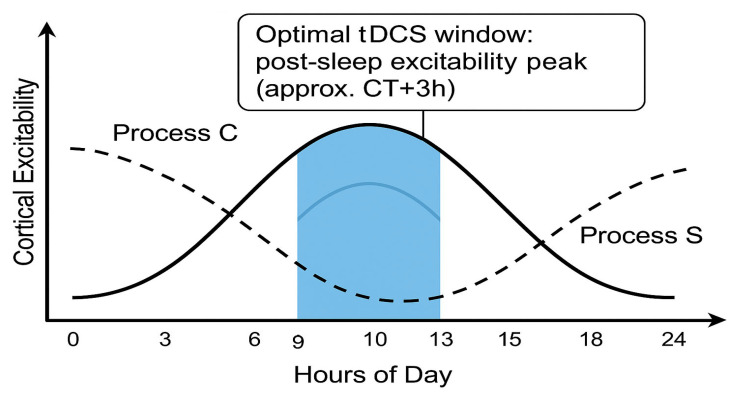
Schematic illustration of the optimal circadian window for tDCS aligned with healthy biological rhythms (Process C and Process S). Created using ChatGPT-4.5 “Orion”.

In summary, tDCS holds great promise for enhancing cognitive performance, and its effectiveness can be further optimised by considering the influence of circadian rhythms on cognitive functions. Tailoring tDCS interventions to an individual’s chronotype and the optimal circadian phase can maximise the benefits of brain stimulation, leading to more robust and consistent outcomes. A summary of this section is provided in Table 1. Schematic illustration of the optimal circadian window for tDCS aligned with healthy biological rhythms is presented on Figure 1.

## 4. The Influence of the Circadian Rhythm on Cortical Excitability and Synaptic Plasticity in Animal and Human Studies

### 4.1. Animal Studies

The circadian rhythm, often referred to as the internal 24 h biological clock, influences various physiological processes in all living organisms, including the alternation between rest and activity. In eukaryotic cells, the circadian clock is driven by a series of transcription and post-transcriptional events, collectively creating a molecular clock with a period of approximately 24 h [97]. This intrinsic clock is coupled to cellular functions through the activation of molecular effectors, effectively translating timekeeping into changes in cell state [97]. One such effector is the mechanistic/mammalian Target of Rapamycin protein (mTOR), a crucial protein kinase that plays a fundamental role in synaptic plasticity, neuronal circuit formation and maintenance, experience-dependent synaptic plasticity, learning, and memory [122].

The interaction between the circadian clock and mTOR involves a complex regulatory genetic loop, like a major negative feedback loop, which is centred on the rhythmic regulation of period (per) and cryptochrome (cry) genes, with elements of the circadian clock directly interacting with the mTOR pathway [123]. The circadian regulation of mTOR expression and ribosome biogenesis, as well as the involvement of the clock genes PER and BMAL1, influence the activity of mTOR [123]. Disruptions in the circadian clock can lead to abnormal mTOR activation, affecting various neuronal processes and functions, as implicated in cognitive deficits [124], synaptic plasticity defects [97], and epilepsy [125].

The primary intersection of the circadian rhythm with cellular processes occurs within the suprachiasmatic nucleus (SCN), a small region in the hypothalamus. In the SCN, light-dependent signals from the retina entrain the autonomous clock to the light cycle [123]. The SCN acts as the central pacemaker, sending timekeeping signals to the entire brain and body through synaptic transmission and hormone secretion [97]. Additionally, circadian regulation of mTOR activity has been observed in the SCN. Exposure to light during the subjective dark period leads to the activation of mTOR, effectively anticipating the original circadian cycle. This bidirectional relationship between mTOR and the circadian clock helps in realigning the cell-autonomous clock to the day-night cycle [97].

The influence of the circadian clock on cortical excitability and synaptic plasticity is evident in the modulation of long-term potentiation (LTP) and memory formation [97]. Studies in rodents have shown that hippocampal LTP is affected by the time of day, with conflicting reports on whether LTP is stronger during the day [126] or at night [127]. The circadian regulation of mTOR and other signalling pathways, such as ERK, plays a role in this modulation of synaptic plasticity. Recent findings suggest that the circadian activation of mTOR is linked to intracellular ion homeostasis, influencing neuronal excitability. Changes in intracellular Cl–concentration, driven by circadian mTOR activation, may impact synaptic strength and the amplitude of action potentials [97]. The circadian regulation of mTOR is likely to contribute to the modulation of LTP and the consolidation of synaptic plasticity. Similar to mTOR, the ERK pathway is regulated by the circadian clock. ERK phosphorylation in cortical neurons fluctuates with wake and sleep cycles, and disruptions in ERK signalling can alter sleep duration and prevent neuronal plasticity triggered by environmental factors. The ERK pathway correlates waking experiences with synaptic plasticity, and its cyclic activation is associated with the expression of activity-regulated genes while awake. During sleep, ERK signalling contributes to synaptic homeostasis, renormalising synaptic strength. ERK activation is critical for various forms of synaptic plasticity, including hippocampal LTP, and is implicated in the consolidation of ocular dominance plasticity in the visual cortex [97].

Overall, the impact of the circadian rhythm on cortical excitability and synaptic plasticity highlights the importance of understanding the complex interactions between circadian biology, mTOR, and other signalling pathways in maintaining brain function and cognitive processes [97]. Disruptions to this delicate balance can give rise to various neurological disorders and cognitive deficits, making circadian biology an essential area of study for improving brain health and performance.

Given the influence of the circadian rhythm on cortical excitability and synaptic plasticity, it is reasonable to consider its potential impact on tDCS. The following section discusses relevant evidence from human studies that shows the huge impact of circadian rhythm-related factors on human cortical excitability and neuroplasticity.

### 4.2. Human Studies

In this section, we discuss studies showing how circadian factors such as sleep pressure, chronotype, and time of day have an influence on cortical excitability and brain plasticity. While there is no experimental demonstration yet of how cortical excitability impacts tDCS outcomes, research has investigated this aspect using transcranial magnetic stimulation (TMS), an alternative non-invasive brain stimulation method.

A study [128] that integrated TMS with EEG has revealed that the effectiveness of TMS is linked to the brain’s existing state. The study delved into the impact of variations in rhythmic brain activity, particularly within the alpha-frequency band (8–14 Hz), on the results of brain stimulation. Reduced activity in the alpha band is believed to signify increased cortical excitability, while heightened activity may indicate cortical idling or inhibition, leading to decreased excitability. The researchers established a relationship between the alpha-band’s resting oscillatory activity in the occipital brain region and the stimulation intensity required to evoke visual perceptions known as phosphenes. They observed a correlation suggesting that TMS-induced synchronised bursts of activity are more likely to yield perceptual effects when cortical activity is less synchronised, as opposed to when it is highly synchronised with alpha-oscillations. This is because the impact of synchronised neural activity triggered by TMS is more noticeable when alpha-activity is low compared to when it is high.

Zrenner et al. [129] investigated how the M-rhythm phase affects corticospinal excitability using EEG-triggered TMS on M1. High excitability at the M-rhythm’s negative peak led to an LTP-like enhancement in corticospinal excitability. Administering TMS during low-excitability or arbitrary phases had no significant effect. Of the participants, 91.3% showed an LTP-like surge during the high-excitability phase. This surpasses results from studies without EEG data. The LTP-like surge during the negative peak was substantial (Cohen’s d = 1.32).

As noted earlier, sleep deprivation, an important circadian factor, significantly impaired human cognition following one night of sleep deprivation. Here we discuss the physiological results. The research observed the corticospinal and intracortical excitability of the motor cortex in sessions labelled as ‘sufficient sleep’ and ‘sleep deprivation’, using a range of TMS protocols. Measures such as the input–output curve (I-O curve) and intracortical facilitation (ICF) were, respectively, employed to assess overall corticospinal excitability and cortical facilitation. Additionally, cortical inhibition protocols, including short-interval cortical inhibition (SICI), I-wave facilitation, and short-latency afferent inhibition (SAI), were utilised. Each stimulation lasted 7 min. The results suggested that sleep deprivation increased cortical excitability by enhancing glutamate-related cortical facilitation and reducing or reversing GABAergic cortical inhibition. Furthermore, during sleep deprivation, induced LTP-like plasticity (anodal) from tDCS was negated, while inhibitory LTD-like plasticity (cathodal) switched to excitatory LTP-like plasticity. This alteration was linked to a rise in EEG theta oscillations due to sleep pressure, a phenomenon absent in their previous study where they explored the effects of the chronotype-aligned (phase-aligned) peak time of day [57]. The research also illustrated that various cognitive functions, such as learning, memory consolidation, working memory, and attention, all reliant on cortical excitability, were impaired following sleep deprivation.

tDCS during the recovery window may actively accelerate the re-entrainment of cortical and circadian dynamics. Two studies by McIntire et al. showed that placing anodal tDCS over left DLPFC restored psychomotor vigilance for ≥3 h, whereas the sham had no effect [130,131]. These data suggest that the first 1–3 h of wakefulness following recovery sleep constitute a high-leverage period: cortical excitability is still up-scaled, yet homeostatic sleep pressure has been partially relieved, allowing tDCS-induced plasticity to “reset” clock-controlled neural circuits. Clinically, scheduling tDCS sessions on the morning after a consolidated recovery night (or even a strategic 90 min nap) could both enhance therapeutic efficacy and promote earlier circadian phase-angle realignment—complementing the health-protective rationale for morning stimulation.

Multiple mechanisms explain how sleep deprivation alters cortical excitability and tDCS-induced plasticity. Sleep loss leads to heightened glutamatergic activity and reduced GABAergic inhibition, tipping the excitatory/inhibitory balance toward hyperexcitability. This in turn can occlude further plasticity: after 24 h of wakefulness, anodal tDCS no longer induces LTP-like gains, and cathodal tDCS can paradoxically produce excitatory (LTP-like) effects instead of the usual inhibition. A study by Kuhn et al. demonstrated exactly these changes (enhanced facilitation, reduced inhibition, and reversed rTMS effects under sleep deprivation) [32]. There was also a rise in EEG theta power (a marker of high sleep pressure [132,133,134,135,136,137,138,139,140]) observed in the same study. These findings align with the Synaptic Homeostasis Hypothesis: extended wakefulness ‘saturates’ cortical synapses, resulting in globally up-scaled excitability and diminished capacity for further potentiation. Indeed, Vyazovskiy et al. showed in rodents that markers of synaptic strength (e.g., cortical LTP slope and AMPA receptor levels) increase after prolonged wakefulness and then decline after sleep [141], supporting the idea of sleep-dependent synaptic down-scaling. In humans, TMS-EEG and TMS-motor studies confirm that cortical excitability steadily rises with time awake and is reset by sleep [39]. For instance, the amplitude of TMS-evoked EEG responses grows from morning to evening and after a night without sleep; then it reverts to baseline after recovery sleep. Likewise, EEG slow-wave (theta) activity—a proxy for homeostatic sleep drive—accumulates with wakefulness and correlates with increased cortical excitability.

Beyond total-sleep-deprivation paradigms, recent closed-loop protocols now allow researchers to down-regulate slow-wave sleep (SWS) without shortening total sleep time. Fehér et al. suppressed SWS by ≈30% in healthy adults using an automated, frontal-template auditory closed loop that delivered stochastic pink-noise bursts whenever online EEG matched a SWS signature. The intervention preserved total sleep time yet shifted residual slow-wave activity to the late night and abolished the customary overnight fall in waking theta power, a marker of synaptic down-scaling [142]. The study therefore offers a powerful human model of ‘selective Process S insufficiency’ and predicts that tDCS sessions scheduled after such a night—or after naturally fragmented SWS—will confront elevated baseline excitability akin to partial sleep deprivation.

Overall, sleep deprivation can enhance cortical excitability by boosting glutamate-related facilitation and reducing GABAergic inhibition. This increased excitability can obstruct the formation of long-term potentiation (LTP)-like plasticity after anodal tDCS. This hindrance may stem from alterations in the balance between excitatory and inhibitory neurotransmission, affecting the threshold for LTP initiation. Furthermore, sleep deprivation can convert the inhibitory long-term depression (LTD)-like plasticity associated with cathodal tDCS into an excitatory LTP-like form. This shift could be a consequence of changes in GABAergic inhibition and glutamate-related facilitation. The alterations in plasticity induced by cathodal tDCS could contribute to impaired motor learning as the typical inhibitory response is reversed in sleep deprivation conditions.

One individual difference in circadian preferences is “chronotype”, which briefly refers to the natural tendency toward morningness or eveningness. In Salehinejad et al. [57], they investigated the effects of chronotype and circadian-preferred and non-preferred times of day on tDCS-induced LTP/LTD-like plasticity, motor intracortical facilitation/inhibition, measured by TMS, and associated cognitive functions (motor learning). The physiological results of this study suggest that the chronotype-aligned (phase-aligned) peak time of a day was associated with enhanced corticospinal excitability, cortical facilitation, and reduced cortical inhibition, compared to the circadian non-preferred time. Specifically, the results demonstrated that anodal tDCS-induced LTP-like plasticity and cathodal tDCS-induced LTD-like plasticity are more pronounced at the chronotype-aligned peak time for both ECs and LCs. This shows that performing stimulation during the preferred time of day, when cortical excitability is greater, produces better results. As mentioned in the previous section, enhanced plasticity was associated with outperformance in motor learning task performance (behavioural counterpart of plasticity) and working memory and attention, which are linked to enhanced cortical excitability (prominent cortical facilitation, diminished cortical inhibition).

To maximise the benefits of tDCS, it would be important to consider the circadian phase of the individual and the targeted brain region. Chronobiological considerations could potentially optimise the timing and duration of tDCS sessions to align with periods of heightened cortical excitability and synaptic plasticity. Neurophysiological findings further support this perspective. A review by Yamada and Sumiyoshi [143] emphasised that tDCS-induced cortical plasticity is mediated by LTP and LTD mechanisms. They highlight how tDCS affects intracellular signalling cascades, particularly through NMDA receptor activation and calcium-dependent plasticity pathways, which are crucial for synaptic modification. These insights suggest that variations in cortical excitability across the circadian cycle could modulate LTP and LTD induction, thereby influencing the effectiveness of tDCS interventions. However, it is essential to acknowledge that the relationship between circadian rhythm, cortical excitability, and tDCS is complex and warrants further investigation. Therefore, future research should focus on understanding the precise mechanisms by which the circadian clock modulates cortical excitability and how it impacts the effects of tDCS.

### 4.3. Meta-Analytic Evidence for Sleep-Pressure-Dependent Shifts in Cortical Excitability

A recent quantitative synthesis powerfully reinforces the single-experiment evidence reviewed in the previous section. Zhang and colleagues [144] pooled every TMS study that measured cortical excitability immediately after a minimum of twenty-four hours of continuous wakefulness in healthy adults. Fourteen experiments, comprising 217 participants, using both EMG and EEG read-outs, converged on the same physiological picture: one night of total sleep deprivation collapses GABA_A-mediated short-interval intracortical inhibition, inflates the amplitude and initial slope of the TMS-evoked potential, and leaves indices such as intracortical facilitation and resting motor threshold numerically higher, though not significantly. Effect sizes are strikingly large (g ≈ 1–2), and a meta-regression showed that the youngest samples display the greatest loss of inhibition, hinting at an age-sensitive vulnerability of cortical homeostasis [144]. The authors interpret these findings as direct evidence that high sleep pressure (Process S) shifts the cortex into a hyper-excitable, plasticity-saturated state; in other words, they provide a quantitative backbone for the Synaptic Homeostasis Hypothesis and for the well-known failure of anodal tDCS to induce LTP-like after-effects under sleep-deprived conditions, first demonstrated by Kuhn et al. [32].

Longitudinal work maps the build-up of this hyper-excitability across the waking day. Huber et al. [39] sampled TMS-EEG responses every two hours for nineteen hours and showed a near-linear rise in TEP slope that reset after one night of recovery sleep. A threshold-tracking study by Mroczek and co-workers extended the picture to GABAB and axonal compartments, demonstrating a progressive drop in short-latency afferent inhibition together with a steeper stimulus–response curve [145]. By contrast, forced-desynchrony experiments that isolate the circadian oscillator in the absence of accumulating sleep pressure—most notably Ly et al.—produce only modest, biphasic swings in excitability, peaking in the normal waking day [78]. Taken together, these lines of evidence show that Process S exerts a quantitatively larger and more monotonic influence on cortical excitability than Process C and that the two processes must therefore be disentangled whenever the efficacy of transcranial stimulation is evaluated.

For the present review, the Zhang et al. [144] meta-analysis and the longitudinal studies form a crucial bridge between our mechanistic discussion of synaptic down-scaling and the practical guidelines we advance later. They demonstrate, with unprecedented statistical power, that the same neurochemical signature—elevated glutamatergic drive and weakened GABAergic inhibition—that boosts baseline motor-cortex output after sleep deprivation simultaneously abolishes or even reverses the canonical polarity-specific effects of tDCS. In other words, stimulation delivered when Process S is high operates on an already potentiated network and can no longer induce orderly LTP or LTD; stimulation delivered after adequate sleep, or at the individual’s circadian-preferred time when sleep pressure is minimal, interacts with a cortex that is once again “plasticity-ready.”

Because this synthesis both quantifies the magnitude of Process S effects and resolves earlier single-study inconsistencies, it fits most naturally at the end of our human-evidence section, immediately after the description of individual sleep-deprivation experiments. Placed there, it provides a seamless transition to the neurotransmitter section that follows: the meta-analytic reduction in SICI and rise in TEP slope are the macroscopic footprints of the glutamate–GABA imbalance we discuss next. Moreover, the pooled data supply numerical benchmarks that can be contrasted with the smaller circadian-only effects, sharpening our later recommendations on study design and clinical scheduling. Finally, they underscore a central message of this paper—that truly personalised, time-sensitive tDCS must account for both the phase of the circadian clock and the level of sleep pressure if it is to avoid the plasticity saturation that currently blunts therapeutic impact.

### 4.4. Borbély’s Two-Process Model and Cortical Excitability

Early EEG telemetry in animals established that SWA behaves exactly as Borbély’s Process S, while REM/NREM timing follows a circadian trajectory; the synthesis became the original two-process model and its later updates. What remained unclear in the 1980s was whether moment-to-moment cortical responsiveness in the awake brain would exhibit the same dual control.

That question was tackled once non-invasive TMS made it possible to probe the human cortex repeatedly across day-long protocols. The pioneering 24 h sleep-deprivation study by Civardi et al. showed that, after one night awake, paired-pulse TMS revealed a marked collapse of both short-interval intracortical inhibition and facilitation—an overall shift in the excitation–inhibition balance toward greater net gain [146]. Six years later, De Gennaro et al. combined TMS with high-density EEG and quantified this shift against classic EEG markers of sleepiness (theta/delta build-up); excitability tracked those markers almost one-to-one across 40 h awake [147]. These experiments provided the first direct evidence that Process S is mirrored by a monotonic increase in cortical gain.

The next step was to assess whether recovery sleep resets that gain—and here Huber et al. [39] delivered a decisive answer. Using TMS-evoked potentials (TEPs), they found that the slope and amplitude of the first cortical response component rose steadily from morning to evening, climbed further after a total-sleep-deprivation night, and snapped back to baseline after a single night of recovery sleep, mapping perfectly onto the exponential build-up and decay of Process S [39].

However, Borbély’s model also predicts a time-of-day modulation independent of prior wake. To isolate Process C, Ly et al. [78] placed participants in a 29 h constant-routine protocol, aligning data to individual melatonin phase. Both TEP amplitude and slope waxed and waned sinusoidally, peaking in the biological evening “wake-maintenance zone” and bottoming out in the early-morning “sleep-promotion zone”. Crucially, a sine wave (circadian) term explained variance above and beyond a linear (homeostatic) term, confirming a bona fide circadian oscillation of cortical excitability [78].

Rodent work provides a mechanistic backdrop: patch-clamp recordings show that firing probability and membrane conductances cycle with the molecular clock via mTOR-gated translation programmes [97], while multi-unit implants reveal a steady rise in cortical firing during enforced wake that is renormalised in early sleep—the synaptic-homeostasis pattern [141]. Thus, at the cellular level, Process S corresponds to cumulative synaptic potentiation and elevated firing, whereas Process C gates this baseline through a clock-dependent modulation of intrinsic and network conductances.

Back to humans, two large-sample studies have put the dual-drive prediction to stringent statistical tests. Chia et al. repeated five TMS protocols through 34 h awake, showing that a multivariate excitability signature could quantitatively decode subjective sleepiness even after the melatonin phase was regressed out—strong evidence that the homeostatic component survives circadian adjustment [148]. Conversely, fitting both drives simultaneously produced the best model fit in a forced-desynchrony-style re-analysis of the Ly et al. [78] dataset, underscoring additivity rather than redundancy between S and C.

Not every metric is equally sensitive, and null results are informative: a comprehensive threshold-tracking study by Mroczek et al. found that most conventional excitability measures were flat after 24 h awake except for a subtle cholinergic short-latency afferent-inhibition change [145]. Together with sex-specific effects in earlier work, this heterogeneity indicates that the imprint of Processes S and C is strongest in early TEP dynamics and in paired-pulse protocols probing GABA-ergic circuits—parameters that directly reflect synaptic efficacy and membrane excitability.

### 4.5. Where Transcranial Direct Current Stimulation Sits in the Two-Process Theory?

Everything we just traced with TMS-EEG—the steady homeostatic rise in cortical gain across wake, and the sinusoidal circadian swing that gates that gain—reappears when we switch from measuring excitability to modulating it with tDCS. This is more than a curiosity: if the membrane is already biased upward by Process S or by the evening crest of Process C, the extra electric field delivered by tDCS lands on a fundamentally different physiological baseline, changing both the direction and magnitude of its after-effects.

The first hint came from the classic paper by Marshall et al., who delivered slow-oscillatory tDCS (0.75 Hz) during the first SWS cycle, when sleep pressure is maximal, and found that it amplified slow waves and consolidated declarative memories [149]. The stimulation did not *create* SWA; it boosted a network that was already primed by high Process S. The converse holds for wakefulness. In one motor-cortex study, a single night of total sleep deprivation pushed baseline MEP amplitudes up but—crucially—abolished the usual anodal LTP-like plasticity and even inverted cathodal LTD-like after-effects [118]. In other words, when synapses are already saturated by prolonged wakefulness, tDCS cannot drive them any higher and may paradoxically excite them when it would normally suppress. That “ceiling effect” is the behavioural equivalent of the TMS gain saturation we found after 40 h awake.

If sleep pressure is kept low but the clock is shifted, tDCS shows a different face. Salehinejad et al. [57] tested “larks” and “owls” at both 08:00 and 19:00. Anodal and cathodal stimulation produced larger, longer-lasting plasticity when applied at each participant’s circadian-preferred time—morning for larks, evening for owls—even though everyone was equally rested. TMS measures collected in the same sessions confirmed a parallel rhythm in glutamatergic facilitation and GABAergic inhibition, exactly matching the Process C swing described earlier [57].

Combining the two drives in a factorial design reveals an almost algebraic pattern of interaction. A 2023 follow-up conference report crossed chronotype (preferred vs. non-preferred) with sleep condition (sleep-deprived vs. rested). The largest potentiation emerged when both drives favoured wakefulness (low S, optimal C), and the smallest emerged when both opposed it (high S, off-peak C) [150].

TMS taught us that cortical gain itself is sculpted by Borbély’s two variables. tDCS now shows that interventions which depend on that gain inherit the same two-process fingerprint: when Process S is high, the membrane is already depolarised and plasticity saturates; when Process C peaks, intrinsic conductances and NMDA gating amplify the same polarisation shift that tDCS tries to impose. The practical upshot is stark: identical currents delivered at different times of day or different sleep-pressure states can yield opposite physiological and behavioural outcomes. For experimental design, this means controlling (or at least logging) time-of-day and sleep history is non-negotiable. For therapeutics—stroke rehabilitation, depression, cognitive ageing—it suggests a simple factor for enhanced efficacy of stimulation after good sleep and in the patient’s circadian “wake-maintenance” phase. In short, tDCS does not just fit into Borbély’s model; it operates through it, turning the abstract S and C curves into controllable parameters for plasticity engineering.

## 5. The Influence of Neurotransmitter Levels

Neurotransmitter systems in the brain are pivotal in shaping neuronal communication and modulating various physiological and cognitive processes. Among these neurotransmitters, glutamate and gamma-aminobutyric acid (GABA) hold particular importance as they exert opposing effects on cortical excitability and are involved in the mechanisms underlying the effects of tDCS.

Glutamate is the brain’s primary excitatory neurotransmitter [151]. It acts on ionotropic and metabotropic receptors, leading to neuron depolarisation, and promoting cortical excitability [152]. When tDCS is applied over the brain, it induces a subthreshold modulation of neuronal membrane potentials. Depending on the polarity of the tDCS electrodes, it can either increase or decrease the excitability of cortical neurons. Anodal tDCS (positive electrode over the target area) is believed to lead to neuronal depolarisation, enhancing cortical excitability, while cathodal tDCS (negative electrode over the target area) induces hyperpolarisation, reducing cortical excitability [153]. Recent evidence supports this excitatory role of tDCS in modulating neurotransmitter levels. A study by Alvarez-Alvarado et al. [154] demonstrated that two weeks of active tDCS paired with cognitive training led to a significant increase in glutamate/glutamine concentrations in the prefrontal cortex. However, no significant changes in GABA levels were observed. These findings provide empirical support for the hypothesis that tDCS enhances neuroplasticity through glutamatergic activity while potentially maintaining inhibitory balance. This suggests that the effects of tDCS on cognitive functions may be primarily driven by increased excitatory signalling rather than reduced inhibition.

GABA is the main inhibitory neurotransmitter in the brain. It binds to GABA receptors, GABA-A and GABA-B, and induces hyperpolarisation of neurons leading to decreased excitability [155]. GABAergic inhibition plays a crucial role in balancing and fine-tuning the overall neuronal activity in the brain. Interestingly, tDCS has been shown to modulate GABAergic activity indirectly. For example, some studies have demonstrated that anodal tDCS can increase GABA levels, which might contribute to its inhibitory effects, paradoxically reducing cortical excitability despite being considered an excitatory technique [156]. The circadian rhythm exerts regulatory control over the release and availability of these neurotransmitters in the brain. Studies have shown time-of-day-dependent fluctuations in the concentrations of glutamate and GABA, with their levels varying across different phases of the circadian cycle [157,158]. These variations can impact the responsiveness of the brain to external stimuli, such as tDCS. In addition to circadian influences, emerging evidence highlights the role of neurotransmitter modulation in tDCS-induced plasticity. Yamada and Sumiyoshi [143] reviewed the biochemical effects of tDCS and found that anodal stimulation enhances excitatory synaptic transmission by increasing glutamate release while simultaneously suppressing GABAergic inhibition. Furthermore, they discuss how tDCS interacts with mono-aminergic systems, modulating dopamine, serotonin, and acetylcholine activity in a region-specific manner. These findings align with the hypothesis that neurotransmitter fluctuations contribute to the variability in tDCS outcomes, reinforcing the importance of personalised approaches based on neurochemical states. During certain times of the day when glutamate levels are naturally higher and GABA levels are relatively lower, the response to anodal tDCS might be more pronounced, as the initial cortical excitability is already elevated. Conversely, during periods when GABA levels are higher and glutamate levels are lower, cathodal tDCS might have a more substantial inhibitory effect, as it aligns with the naturally occurring inhibitory processes in the brain. Furthermore, the influence of the circadian rhythm on neurotransmitter levels can also contribute to inter-individual variability in tDCS responses. Individuals with different circadian preferences (e.g., morning type vs. evening type) may experience different neurotransmitter levels at different times of the day, potentially impacting the effectiveness of tDCS for each individual.

### Translating Neurotransmitter Rhythms into tDCS Protocol Design

In this subsection, we present a speculative proposal—a perspective—translating the circadian rhythms of neurotransmitters into practical tDCS protocol recommendations.

Circadian modulation of glutamate and GABA provides a neurochemical map for deciding when and how to stimulate. In the morning, roughly two to three hours after habitual waketime, extracellular glutamate reaches its daily peak, while GABAergic inhibition is at its minimum. This excitatory bias lowers the threshold for long-term potentiation-like plasticity, so anodal stimulation of 1.5–2 mA for approximately twenty minutes over the task-relevant cortex is most likely to enhance learning, memory, and mood circuits at this time; if pre-session TMS or resting-state EEG indicates already elevated excitability, the current can be reduced to 1 mA to avoid a ceiling effect.

By midafternoon, glutamate levels have partly declined, and GABA has begun to rise, producing a more balanced excitatory–inhibitory milieu. Under these conditions anodal tDCS should be paired with active performance of the cognitive or motor task to allow use-dependent plasticity to compensate for the smaller neurochemical drive; passive stimulation alone may yield only modest gains.

In the late evening and early night, glutamate reaches its trough and GABA its peak as the brain prepares for synaptic downselection and sleep. Routine excitatory stimulation is therefore inadvisable. When clinical need dictates evening treatment—for example, to calm insomnia-related hyperarousal—a brief low-intensity cathodal session can be applied to reduce cortical activity, ideally followed by lights-out within thirty minutes to capitalise on the natural GABA surge.

After acute sleep deprivation, glutamate is pathologically elevated and GABA suppressed, creating a hyper-excitable cortex in which even cathodal stimulation can paradoxically induce potentiation. Excitatory sessions are best postponed until after at least one full recovery night; if stimulation cannot be delayed, both current intensity and duration should be reduced and cortical excitability monitored before and after treatment.

Rule of thumb: Schedule anodal stimulation in the morning for learning, mood, or rehabilitation goals; combine anodal stimulation with active training in the afternoon; reserve low-dose cathodal stimulation for evening sedation; and defer or down-titrate stimulation in the acutely sleep-deprived brain. Aligning polarity, intensity, and timing with the endogenous ebb and flow of glutamatergic excitation and GABAergic inhibition maximises intended plastic changes while minimising counterproductive effects. This neurochemical framing dovetails with the chronotype and phase-based recommendations in Section 6 and Section 8, yielding a coherent, circadian-informed rationale for protocol selection.

## 6. Glucocorticoids and the HPA Axis: A Circadian Gate on Plasticity

Cortisol secretion follows a robust circadian profile, peaking 30–45 min after habitual wake time and falling to a nadir around midnight, with ultradian pulses superimposed on this curve [73,75,159,160,161]. Rising cortisol sharpens alertness but also shifts neuronal gain by enhancing glutamatergic drive and suppressing GABAergic tone; a single 20 mg hydrocortisone bolus can raise motor-cortex excitability within 30 min [162]. Crucially, high morning cortisol impairs the induction of Hebbian plasticity. In a paired-associative-stimulation protocol, Sale et al. showed that corticospinal LTP could be abolished when circulating cortisol was high, and restored when the same experiment was repeated at the low-cortisol evening phase [90].

Given the above, it is not surprising that the efficacy of non-invasive brain stimulation—including tDCS—can vary across the day as these physiological parameters change. There is growing evidence that the brain’s response to tDCS is state-dependent and that time-of-day and associated factors like cortisol levels significantly influence outcomes [163]. Many brain stimulation studies explicitly control for time of day in their protocols, recognising that a morning session opposed to an evening session can yield different results even with identical tDCS parameters. For example, Clow et al. demonstrated that higher cortisol levels predict greater response to an rTMS plasticity protocol in the motor cortex [91]. This implies that, if tDCS is applied when cortisol is at a favourable level for that individual, the induced neuroplastic changes might be larger. On the other hand, Sale et al. (discussed above) showed that artificially elevating cortisol at a normally low-cortisol time can negate induced plasticity [90]. Together, these findings suggest a complex interaction: cortisol can both facilitate and suppress plasticity depending on context, possibly following an optimal range. In practical terms, for tDCS, it means the stimulation’s impact on cortical excitability might be amplified at certain circadian phases and blunted at others due to the hormonal milieu.

Similarly, the circadian variation in cortical excitability and network inhibition could modulate tDCS effects. TMS measures have revealed that corticospinal excitability and intracortical inhibition in M1 decrease progressively over the day (i.e., the cortex becomes less excitable by evening) in the average person. This diurnal drift in baseline excitability could alter how the brain responds to tDCS currents. For instance, if the cortex is less excitable in the evening (as Lang et al. reported [164]), anodal tDCS might have a smaller facilitatory effect at that time unless other factors (such as lowered cortisol or higher temperature) counteract it. In contrast, morning brains might have higher excitability but also higher homeostatic plasticity thresholds due to high cortisol. A narrative review on tDCS variability indeed noted that circadian hormones and brain states are likely contributors to the notorious inter- and intra-individual variability in tDCS outcomes [163]. The authors highlighted that TMS carefully studies time sessions to avoid circadian differences and that tDCS studies should consider doing likewise.

A concrete example of these interactions is seen in studies combining brain stimulation with measurements of cortisol. Preliminary trials have found that tDCS effects on cognitive tasks can differ when applied in the morning vs. evening, and that controlling for cortisol can reduce variability in outcomes. One study on tDCS and stress reported scheduling all sessions between late morning and afternoon “to control for endogenous cortisol activity” [165].

Stress-related cortisol surges interfere with tDCS in the same direction: working-memory gains from left DLPFC anodal tDCS were present only under low self-reported stress and reversed under high stress [166]. Conversely, excitability-enhancing tDCS or high-frequency rTMS over pre-/dorsolateral PFC can suppress the cortisol response to acute psychosocial stress, pointing to a bidirectional relationship [167,168].

Cortisol therefore acts as a third, hormone-based gate that narrows the Process C × Process S plasticity window during the early morning hours and under acute stress. Scheduling stimulation for the late biological morning or early evening not only aligns with optimal circadian arousal but also with falling glucocorticoid tone, maximising the chance of LTP-like after-effects. Future tDCS studies should log salivary cortisol or pharmacologically manipulate the HPA axis to test this prediction directly.

## 7. Circadian Rhythms, Body Temperature, and Possible tDCS Outcomes

Passive whole-body heat stress provides causal evidence that even a modest, short-lived rise in core temperature can prime motor-cortex plasticity in humans. In a crossover design, Littmann and Shields placed participants in a 73 °C low-humidity chamber for 30 min, which lifted tympanic temperature by ≈2 °C—equivalent to a core increase of ≈0.8 °C—and drove heart rate to ~65% of the age-predicted maximum while subjective warmth returned to baseline within 15 min of exit. Resting motor-evoked-potential (MEP) amplitude, mapped at 15 scalp sites 25 min after heating ended, rose by an average 48% compared with a 4% decline during a sham session, with a significant Group × Time interaction (F = 10.23, *p* = 0.01). Six of eleven subjects—five of six males—showed ≥ 49% facilitation, whereas most females did not, yielding a sex-specific Group × Condition effect (*p* = 0.038) [169]. Taken together, these findings imply that the ≈ 0.5 °C endogenous afternoon temperature crest may act as a natural “heat-prime” for excitatory neuromodulation. Aligning anodal tDCS with this thermal peak—or inducing mild pre-heating in patients who cannot exercise—could magnify and prolong after-effects, while morning sessions conducted near the temperature nadir might require higher currents or longer durations to achieve comparable plasticity. Sex hormones appear to modulate the temperature–excitability link, so future tDCS studies should stratify by sex or menstrual phase when adding thermal manipulations.

## 8. Cortisol and Temperature as Modulators in a tDCS “Window of Efficacy”

The converging evidence above leads to the concept of a “window of efficacy” for tDCS—an optimal period in the day when the physiological milieu is most supportive of induced neuroplasticity. Theoretical and empirical bases for this concept come from chronobiology and neuroscience.

Circadian Alignment for Plasticity: As discussed, late afternoon/early evening is characterised by low cortisol and high core body temperature, a state which appears to favour synaptic plasticity. Low cortisol removes the brake on LTP-like mechanisms (demonstrated by PAS being strongest in the evening), while high temperature and arousal maximise neuronal responsiveness. This period often coincides with the peak of cognitive performance for many individuals. Thus, we expect tDCS to be most efficacious when applied in this window, tapping into a brain already tuned for learning and network reconfiguration. In contrast, early morning (high cortisol, low temperature) might be a suboptimal window—the brain may be awake but not yet in a plastic-ready state, and tDCS effects could be smaller or more transient.

Empirical Support for Optimal Timing: A study by Sale et al. effectively demonstrated a circadian modulation of induced plasticity: endogenous low cortisol (evening) was associated with greater motor cortex plastic changes, whereas raising cortisol at that time negated the effect [90]. Similarly, Ly et al., who mapped cortical excitability over 29 h, speculated from their data that there may be optimal times of day for neurorehabilitation approaches or non-invasive neurostimulation, given the circadian swings in excitability [78]. They observed that brain responsivity experienced non-linear changes, dipping and surging with circadian phase, implying that timing stimulation to those dynamics could improve outcomes. In clinical contexts, patients with neurodegenerative diseases often have blunted or shifted rhythms; aligning stimulation with whatever residual rhythms remain (or even pharmacologically shaping them) might expand the window of efficacy.

Theoretical Integration—“Chronotherapy” for Brain Stimulation: In chronobiology, treatments timed to biological rhythms (chronotherapy) can substantially enhance effectiveness. By analogy, tDCS delivered at an individual’s optimal circadian phase could yield stronger and more lasting neuroplastic effects, forming a basis for personalised “brain stimulation chronotherapy.” Cortisol and temperature rhythms are key guides to find this phase. For instance, an ideal scenario might be the following: cortisol has substantially declined from its morning peak (reducing its inhibitory influence on synapses), core temperature and alertness are near their daily maximum, and the person is not yet mentally fatigued from prolonged wakefulness. This likely corresponds to mid-to-late afternoon for a typical diurnal person. Within that window, the ratio of excitatory/inhibitory neurochemicals (e.g., higher BDNF, lower adenosine) is conducive to plastic change. In contrast, outside the window (e.g., very late at night when temperature plummets and melatonin rises), the brain may actively oppose plastic induction as it prepares for sleep—tDCS then might have diminished or unpredictable effects.

Balancing Cortisol’s Effects: It is worth noting that, while low cortisol is generally better for inducing plasticity, some cortisol is still needed for normal function. A flat cortisol rhythm (no clear peak) is associated with fatigue and cognitive dulling. Therefore, the window of efficacy is not about minimising cortisol per se but finding when cortisol is in its optimal range. This could mean after the morning spike has passed but before cortisol becomes so low that energy levels drop. Empirical data hint that mid-day cortisol trough (early afternoon) might coincide with a slight “post-lunch dip” in alertness for some—so the late afternoon when cortisol is low but stable and temperature is high might be ideal. Indeed, PAS and TMS studies show late afternoon/early evening superiority for plasticity, aligning with this reasoning. Therefore, integrating cortisol and temperature rhythms suggests a clear target: schedule stimulation when cortisol is naturally low (preventing hormonal suppression of plasticity) and when temperature/alertness are high (promoting neuronal engagement).

In sum, the circadian timing signal (via cortisol, temperature, etc.) creates shifting windows where the brain is either more amenable or more resistant to external modulation. Theoretical support for a tDCS window of efficacy comes from our growing understanding of how sleep–wake biology gates plasticity—akin to how the brain favours memory consolidation at night and experience-driven plasticity during the day. By treating cortisol and temperature as modulators, we can refine tDCS protocols to hit the brain’s sweet spots for inducing change.

## 9. Individual Variability and Personalised Approaches

To optimise tDCS outcomes, it is essential to adopt personalised approaches that consider an individual’s circadian typology and cognitive patterns. For instance, morning types may benefit from tDCS administered during the early hours when their cognitive functions are naturally enhanced. In contrast, evening types may experience greater cognitive enhancements when stimulated during their peak alertness in the late afternoon or evening. Tailoring tDCS interventions based on an individual’s chronotype and circadian phase can maximise the benefits of stimulation and lead to more robust cognitive improvements, not just in healthy humans but also in patients with neuropsychiatric disorders, as shown in recent works [170,171,172].

In addition to circadian considerations, recent research highlights the importance of combining tDCS with cognitive training to enhance its effects on neuroplasticity. Alvarez-Alvarado and colleagues [154] demonstrated that a two-week intervention of tDCS paired with cognitive training led to increased prefrontal glutamate concentrations, suggesting that the excitatory mechanisms of tDCS may be more effective when stimulation is combined with cognitive tasks that engage targeted brain regions. This supports the notion that time-sensitive and task-specific stimulation protocols could optimise tDCS efficacy, reinforcing the need for individualised intervention strategies.

Incorporating individual differences in circadian typology into tDCS research may also help explain conflicting findings in previous studies. Differences in the optimal timing for stimulation among participants with diverse chronotypes could have contributed to the variability in tDCS outcomes reported in the literature. A recent meta-analysis by Saleh and colleagues [173] aligns with this perspective, showing that tDCS did not significantly improve cognitive outcomes in individuals with mild cognitive impairment (MCI). The authors suggested that individual variability in response to tDCS might stem from differences in chronotype, stimulation timing, and baseline cortical excitability. Notably, many of the failed or mixed trials in MCI employed tDCS sessions scheduled at widely varying times—some mid-morning, others late afternoon or evening—without reference to participants’ individual sleep–wake schedules or internal circadian phase. Such unanchored timing can lead to chronic phase misalignment, whereby stimulation falls during suboptimal windows of low cortical excitability or high homeostatic sleep pressure, undermining plasticity induction. This temporal heterogeneity likely contributes to the high between-study variability observed in the meta-analysis, masking potential benefits when averaged across non-aligned sessions. In contrast, adopting a uniform, circadian-aligned protocol (e.g., wake time + 2–3 h) could reduce this source of noise and reveal more consistent, robust cognitive gains in MCI cohorts. These findings highlight the importance of optimising tDCS parameters based on circadian and individual neurophysiological factors to maximise cognitive benefits. By recognising these individual differences and accounting for them in experimental design, researchers can establish a more comprehensive understanding of tDCS effects and pave the way for personalised brain stimulation protocols. Moreover, wearable devices and mobile apps that track circadian rhythms and sleep patterns have become increasingly available, providing more opportunities for the real-time monitoring of individual circadian phases and performance fluctuations. These technologies can be integrated into tDCS research and clinical practice to optimise stimulation timing and tailor interventions to the circadian profile. For example, over the seven days preceding the first tDCS session, participants will wear a wrist actigraph (30 s epoch resolution) and will maintain a daily sleep diary to capture objective and subjective sleep–wake timing, midsleep, sleep efficiency, and social jet-lag. In parallel, a consumer wrist- or ring-style tracker synced to a smartphone app will continuously upload activity, light exposure, and heart-rate data to the cloud, allowing real-time detection of phase shifts (for example, weekend–weekday drift) and automatic alerts if habitual wake times diverge by more than 30 min. Optional nightly recordings from a home EEG headband can quantify frontal θ/α ratios and resting-state α-peak frequency in the first hour after waking, providing a running index of day-to-day fluctuations in cortical excitability. A one-time Morningness–Eveningness Questionnaire (MEQ) will classify trait chronotype—used only for descriptive stratification, not timing decisions. All data will stream feed into a companion app that computes each individual’s phase-aligned window (habitual wake time + 2–3 h), schedules reminder notifications, and logs actual session start times versus planned ones to support iterative schedule refinement.

While individual chronotypes influence when cognitive performance peaks, a more universally beneficial strategy is to align tDCS administration with biologically optimal circadian phases. In practice, this means scheduling stimulation during morning hours after a full night of sleep, when cortical excitability and alertness are naturally elevated. Delivering tDCS in these high-excitability periods can maximise its neuromodulatory impact, leveraging the brain’s refreshed state upon waking. Importantly, this circadian-aligned approach also promotes healthy sleep–wake patterns. By contrast, offering stimulation at a person’s subjective late-day “peak” (e.g., late evening for an extreme “night owl”) may coincide with a misaligned circadian phase that is suboptimal for plasticity and could reinforce maladaptive scheduling (such as a delayed sleep phase). Chronic circadian misalignment is associated with negative health and mood outcomes—for example, delayed sleep–wake phase disorder raises the risk of depressive symptoms [8], and circadian disruption more broadly is linked to metabolic and cardiovascular impairments [174]. Therefore, unless there is a strong contra-indication, tDCS sessions should default to morning (i.e., circadian-aligned) time slots following sufficient sleep. Such timing takes advantage of the morning excitability peak and supports long-term circadian health, rather than exacerbating irregular rhythms.

## 10. The Impact of tDCS on the Circadian Rhythm—A Bidirectional Relationship?

Although not the focus of the current review, it cannot be ignored that tDCS may influence the circadian rhythm by affecting various parameters of sleep and vigilance. Investigating the influence of bifrontal anodal tDCS on sleepiness and vigilance, Alfonsi et al. [60] revealed a compelling efficacy in reducing physiological sleepiness and preventing vigilance decline. Notably, the impact was more pronounced during the afternoon session, suggesting a possible interaction with circadian influences. Frase et al. [175] examined tDCS effects on total sleep time (TST), unveiling a polarity-specific reduction with bifrontal anodal stimulation. This reduction, not observed in the control group with reversed electrodes, suggests location specificity. Exploratory EEG analyses implicated changes in cortical arousal, emphasising the potential role of tDCS in modulating sleep continuity. In a similar vein, the results of Jung and Jun [176] on patients with chronic insomnia showed tDCS improved TST, sleep latency (SL), and sleep efficiency (SE). McIntire et al. [130,131,177] explored the impact of anodal tDCS on sleep-deprived individuals, comparing its effects with caffeine. Results indicated that tDCS not only prevented vigilance decrement but also demonstrated lasting effects on vigilance, reaction time, and mood. The study further hypothesised and verified that stimulation to specific brain regions could influence arousal and sleep time. Several studies [178,179,180] provided evidence supporting the notion that tDCS may counter cognitive decline induced by circadian rhythm disruption through sleep deprivation. Whether administered during sleep deprivation or after, tDCS consistently exhibited beneficial effects on working memory, attention, response time, and vigilance. Importantly, these effects persisted beyond two hours, showing the enduring impact of tDCS on cognition during sleep deprivation.

## 11. Practical Implications and Future Directions

Incorporating circadian considerations into tDCS research and practice presents exciting opportunities for improving cognitive enhancement and therapeutic applications. To fully harness the potential of circadian-adjusted tDCS, several practical implications should be considered:

Chronobiological assessment: When designing tDCS studies, researchers should consider incorporating chronobiological assessments, such as actigraphy and questionnaires, to identify each participant’s circadian typology and peak performance times. This approach is supported by findings from Xu et al. [111], who reviewed evidence demonstrating that cognitive performance follows a rhythmic pattern throughout the day. They suggest that scheduling cognitive tasks and interventions during an individual’s optimal circadian window can significantly enhance performance. These insights align with the growing emphasis on personalised brain stimulation approaches that take circadian influences into account when optimising neuroplasticity and cognitive outcomes.

Personalised stimulation protocols: Tailoring tDCS protocols based on an individual’s circadian phase and cognitive performance patterns allows for the development of personalised approaches that align with periods of heightened cognitive function, thereby enhancing the overall effectiveness of stimulation, as shown in a recent study [171]. Empirical evidence suggests that pairing tDCS with cognitive training may further enhance neuroplasticity. A study by Alvarez-Alvarado et al. [154] demonstrated that a two-week intervention of tDCS combined with cognitive training led to increased glutamate levels in the prefrontal cortex, indicating that stimulation effects can be potentiated when paired with cognitive engagement. These findings suggest that combining neurostimulation with task-based training could be a key factor in optimising clinical interventions, reinforcing the importance of multi-modal approaches to enhance cognitive and neurophysiological outcomes.

Sleep optimisation: Recognising the close relationship between circadian rhythms and sleep–wake cycles, integrating sleep optimisation strategies alongside tDCS interventions may further enhance cognitive outcomes and neuroplasticity.

Clinical applications: Exploring the application of circadian-adjusted tDCS in a clinical setting may offer new therapeutic opportunities for neurological and psychiatric disorders. Optimising tDCS timing based on an individual’s circadian phase may improve treatment outcomes and enhance patient responses to brain stimulation, as shown in some studies [170,171,172]. These adjustments are crucial given the inconsistent findings in tDCS research. Circadian timing is not merely a methodological detail; it can determine whether neuromodulation reinforces or counteracts the pathophysiology of rhythm-sensitive disorders. Morning, phase-aligned stimulation—defined here as the first two to four hours after habitual wake time—offers distinct advantages across several clinical domains. First, mood disorders such as major depression, bipolar illness, obsessive–compulsive disorder, and social anxiety are frequently accompanied by delayed or blunted morning arousal. Prefrontal anodal tDCS is already an accepted adjunctive treatment for these conditions [172]. Delivering stimulation soon after waking takes advantage of the natural surge in cortical excitability and mono-aminergic tone described in mechanistic reviews [143] while simultaneously nudging the sleep–wake cycle earlier. In practice, sessions should be scheduled two to three hours after the patient’s habitual wake time. If actigraphy reveals a marked phase delay, morning tDCS can be paired with bright-light exposure or sleep-advance counselling to consolidate the earlier rhythm. Second, in mild cognitive impairment (MCI) and early Alzheimer’s disease, cognitive alertness is typically best in mid-morning even when the 24 h activity rhythm is fragmented. A recent meta-analysis that found no overall benefit of tDCS in MCI pointed to heterogeneous timing protocols as a major limitation [173]. Anchoring stimulation to the preserved morning “island” of attentional capacity—again, within the first third of the wake day—may allow parietal or temporal tDCS to interact more effectively with task-dependent long-term potentiation, especially when combined with concurrent cognitive training [154]. Third, in sleep–wake and vigilance disorders (insomnia and shift-work fatigue), several studies have shown that bifrontal anodal tDCS administered in the morning or early afternoon reduces subjective sleepiness and improves vigilance, whereas evening sessions can inadvertently delay subsequent sleep. Therefore, stimulation is best anchored to the biological rather than the roster-defined morning: habitual wake time on free days plus two to three hours or, when feasible, dim-light melatonin onset plus three hours. Fourth, chronic pain conditions such as migraine and fibromyalgia display circadian variations in pain thresholds that worsen when rhythms are delayed. Morning motor-cortex anodal tDCS, delivered before the day’s nociceptive peak, may raise endogenous inhibitory tone without reinforcing maladaptive late-night activity. A practical protocol is to administer stimulation soon after waking on at least three non-consecutive days per week while monitoring sleep diaries to ensure sessions do not drift towards evening. Finally, in post-stroke motor rehabilitation, corticospinal excitability rises sharply soon after habitual wake time. Scheduling motor-cortex tDCS at that moment and following it immediately with physiotherapy can leverage Hebbian principles to strengthen relearning—an approach supported by multiple rehabilitation trials that report larger gains when therapy is front-loaded in the daily schedule [153].

These examples highlight a consistent operational guideline: identify each patient’s habitual wake time using actigraphy or sleep diaries, add two to four hours, and designate this window as the optimal period for stimulation aligned with the circadian phase. Adjustments should be made only when disorder-specific symptoms dictate otherwise. By mapping stimulation onto the underlying circadian biology of each condition, clinicians can maximise tDCS efficacy while avoiding the inadvertent reinforcement of maladaptive phase delays, thus integrating neuromodulation with broader chronotherapeutic strategies.

Combinations with cognitive training: Combining tDCS with cognitive training paradigms during specific circadian phases may amplify cognitive gains and promote skill acquisition in learning and memory tasks.

Consider salivary cortisol or temperature monitoring for precise alignment: In research settings or critical therapeutic applications, one can monitor the individual’s circadian phase via markers like salivary cortisol profiles or continuous core body temperature. Cortisol sampling (e.g., measuring the cortisol awakening response and its decline) is a reliable way to gauge the internal clock’s phase and amplitude. Likewise, tracking oral or tympanic temperature over the day can indicate the timing of that person’s temperature peak. Using these measures, practitioners can align tDCS sessions with the period of lowest cortisol and/or highest body temperature for that individual. As Gobbo and colleagues noted, salivary cortisol monitoring could allow for convenient characterisation of the individual chronotype, and body temperature measurements may also be used to track the endogenous rhythm [80]. In practice, if a patient’s cortisol nadir occurs at 5 PM, this may represent an optimal time for stimulation.

Our review highlights that both circadian phase and sleep history influence tDCS outcomes. A late-day tDCS session occurs at a different circadian time and after many hours of wakefulness (high Process S) relative to a morning session. Thus, an observed “time-of-day” difference might partly reflect acute sleep pressure rather than intrinsic circadian variation. Future studies should control sleep–wake schedules or use protocols (e.g., forced desynchrony) to isolate these factors. Considering Borbély’s model, an optimal tDCS intervention might need to account for both the client’s circadian phase and their accumulated sleep pressure.

Practical guidelines for optimising tDCS based on circadian considerations are presented in Table 2. Table 3 illustrates the health and efficacy benefits of delivering tDCS in a circadian-aligned window versus misaligned timing.

### Operational Details of the Chronobiological tDCS Framework

To make the conceptual workflow actionable, we now detail (i) how chronobiological data are captured and interpreted, (ii) how stimulation windows are scheduled, and (iii) what success metrics are recommended.

Step 1—Baseline chronobiological assessment (Days –7 to 0)

- Actigraphy (≥7 consecutive days; wrist-worn; 30 s epoch resolution) to extract sleep onset/offset, midsleep, and social jet-lag.
- Sleep diary (Karolinska or Consensus diary) to corroborate actigraphy.
- Morningness–Eveningness Questionnaire (MEQ) to classify chronotype but not to dictate stimulation timing.
- Optional EEG (home dry-electrode headband): Nightly frontal θ/α ratio and daytime resting-state α peak to index circadian sleep pressure and cortical excitability.

Step 2—Deriving the optimal stimulation window (Day 0)

- Compute the individual’s average mid-sleep on free days corrected for sleep debt (MSF) as a phase marker.
- Define CT 0 (circadian time zero) as MSF—3 h; morning cortical-excitability peaks typically occur at CT +2 to +4 h.
- Default rule: Schedule tDCS at CT +3 h (≈2–3 h after habitual wake time).
- If objective EEG shows a delayed α-peak (>CT +4 h), shift session earlier by 30 min blocks until α-peak minus 30 min is reached.

Step 3—Stimulation protocol

- Montage: Target-specific (e.g., left DLPFC anode/right supra-orbital cathode for cognitive tasks).
- Intensity/duration: 1.5–2 mA × 20 min (ramp 30 s).
- Contra-indicator: If the participant slept < 6 h the previous night, postpone by 24 h to preserve the “well-rested” criterion.

Step 4—Outcome evaluation (pre-, post-, 24 h, 1 week)

- Neurophysiology: TMS-MEP I/O curve slope; resting-state EEG β-band power.
- Cognition: Three-back accuracy (working memory), Psychomotor Vigilance Task median RT.
- Sleep and circadian: Actigraphy-derived sleep efficiency, phase-angle difference (habitual wake time—tDCS start).
- Subjective: Karolinska Sleepiness Scale, tDCS side-effect questionnaire.

Decision rule for iteration: If post-session cortical excitability ↑ ≥ 15% and there are no adverse events, retain timing; otherwise, advance the session by –30 min the following week.

## 12. Conclusions

In this review, we examined the bidirectional relationship between circadian rhythms and tDCS, exploring how circadian factors (chronotype, sleep pressure, time of day, neurotransmitter fluctuations) influence tDCS outcomes and how tDCS affects circadian processes (sleep, vigilance). Evidence suggests circadian rhythms modulate cortical excitability and synaptic plasticity via dynamic shifts in glutamatergic facilitation, GABAergic inhibition, and molecular pathways. Anodal tDCS is more effective when cortical excitability is optimal (e.g., preferred circadian time, after sufficient sleep), promoting LTP-like plasticity and cognitive gains. Conversely, reduced excitability (e.g., non-preferred circadian phase, sleep deprivation) can diminish or reverse tDCS effects, highlighting the importance of state-dependent tDCS. Personalised approaches, accounting for chronotype, are crucial. Morning types may benefit most from early-day stimulation, while evening types might respond better to afternoon/evening stimulation, aligning with individual peaks in excitability and performance. Combining tDCS with sleep optimisation further enhances neuroplasticity. Preliminary studies indicate that tDCS can modulate vigilance and sleep, suggesting its potential as a therapy for circadian misalignment or disorders. Future research should include large-scale, longitudinal studies integrating chronobiological assessments, wearable sleep tracking, and individualised tDCS schedules to optimise circadian-timed interventions for both healthy and clinical populations, advancing personalised neuromodulation. In conclusion, circadian rhythms significantly shape tDCS outcomes, and tDCS can affect circadian regulation. Recognising this interplay enables more precise, time-specific, and personalised interventions. By considering chronotype, time-of-day, and sleep–wake cycles, tDCS protocols can be enhanced for more consistent and effective cognitive improvements and therapeutic benefits.

Beyond cognitive and therapeutic efficacy, adopting a circadian-aligned schedule for tDCS may itself help offset the well-documented health risks of chronic phase misalignment. Late chronotype, rotating shift work, and delayed sleep–wake phase disorders are associated with higher rates of depression, certain cancers, metabolic dysregulation, and chronic pain-related inflammation. Delivering stimulation in the early morning—roughly two to three hours after habitual wake time—can acutely advance melatonin onset, reinforce a more stable light-entrained rhythm, and reduce homeostatic sleep pressure. By nudging the circadian phase earlier while simultaneously modulating cortical excitability, morning tDCS therefore acts as a dual-action intervention: it enhances neuroplasticity and cognition and helps correct the misalignment that underpins mood, metabolic, and pain vulnerabilities. Integrating this chrono-therapeutic perspective into clinical protocols positions tDCS as a practical tool not only for symptom control but also for long-term health promotion in rhythm-sensitive populations.

## Figures and Tables

**Table 1:** Summary of circadian influences on cognitive functions and tDCS effects.

tDCS Implication	Circadian Influence	Peak Performance Time	Cognitive Function	
Morning tDCS may enhance attention-biased tasks	Higher cortical excitability and fast reaction times	Morning	Attention and Alertness	1
Midday tDCS could optimise working memory improvements	Glutamate levels peak, enhancing short-term information processing	Late Morning to Afternoon	Working Memory	2
Afternoon tDCS may maximise executive function gains	Enhanced prefrontal cortex function for problem-solving	Afternoon	Executive Function	3
Late afternoon stimulation may enhance motor skill learning	Increased plasticity in motor regions, optimal for skill learning	Late Afternoon to Evening	Motor Learning	4
Evening tDCS could facilitate memory consolidation	Higher hippocampal activity, improved memory retention	Evening to Night	Memory Consolidation	5

**Table 2:** Practical guidelines for optimising tDCS based on circadian considerations.

Factor	Consideration	Practical Recommendation
Chronotype	Stimulation during morning circadian phases enhances efficacy for most individuals post-sleep recovery	Schedule tDCS ~2–3 h after habitual wake time (CT + 3 h) to align with the morning excitability peak
Time of Day	Aligning tDCS with cognitive peaks enhances effectiveness	Time sessions when the targeted cognitive domain naturally peaks
Sleep Deprivation	Sleep deprivation alters cortical excitability and can reverse tDCS effects	Avoid tDCS after < 6 h of sleep; if so, postpone until after a full night’s recovery sleep
Neurotransmitter Levels	tDCS interacts with glutamate/GABA balance; optimal timing depends on current neurochemical milieu	Leverage EEG or neuroimaging to identify when glutamate/GABA ratios favour LTP-like plasticity
Task-Specific Stimulation	Stimulation should target the cognitive function naturally peaking at that circadian phase	Combine tDCS with task-specific training during the corresponding peak window for maximal transfer

**Table 3:** Health and efficacy benefits of delivering tDCS in a circadian-aligned window versus misaligned timing.

Misaligned tDCS	Circadian-Aligned tDCS	Outcome Measure	Benefit Domain
Blunted or reversed plasticity; anodal fails to induce LTP, cathodal may paradoxically induce facilitation	Robust polarity-specific LTP/LTD, prolonged after-effects (up to ≥90 min)	Magnitude and duration of LTP-like after-effects	Neuroplasticity
Impaired learning, memory, and attentional performance after misaligned/timed poorly relative to chronotype	Faster motor skill acquisition; ↑ accuracy and speed in WM and attention tasks	Motor learning (SRTT), working memory (3-back), attention (Stroop)	Cognitive Performance
No change or further phase delays; fragmented sleep persists	Phase-angle misalignment (∼–45 min earlier melatonin onset), ↑ nightly sleep efficiency	Melatonin onset, sleep efficiency	Sleep and Circadian Health
No improvement or potential worsening in mood/inflammation	Self-rated depressive/anxiety scores; ↓ systemic inflammation markers (e.g., IL-6)	Depressive/anxiety symptoms, inflammatory markers	Mood and Disease Risk
High inter-subject and inter-session variability; poor reproducibility	Variability across sessions; more predictable polarity-specific effects	Inter-session variability and reproducibility of tDCS outcomes	Therapeutic Consistency

## Data Availability

No new data were created or analysed in this study.
